# Diagnosis and treatment of patients with suspected mucinous cystic neoplasms of the liver: a retrospective cohort study

**DOI:** 10.1007/s00423-024-03246-7

**Published:** 2024-02-17

**Authors:** Alicia Furumaya, Hannah H. Schulz, Joanne Verheij, R. Bart Takkenberg, Marc G. Besselink, Geert Kazemier, Joris I. Erdmann, Otto M. van Delden

**Affiliations:** 1 Amsterdam UMC, Department of Surgery, Location University of Amsterdam, Amsterdam, Netherlands; 2 Amsterdam Gastroenterology Endocrinology Metabolism, Amsterdam, Netherlands; 3 Amsterdam UMC, Department of Radiology, Location University of Amsterdam, De Boelelaan 1117, 1081 HV Amsterdam, Netherlands; 4 Amsterdam UMC, Department of Pathology, Location University of Amsterdam, Amsterdam, Netherlands; 5 Amsterdam UMC, Department of Gastroenterology and Hepatology, Location University of Amsterdam, Amsterdam, Netherlands; 6 Department of Surgery, Amsterdam UMC, Location Vrije Universiteit Amsterdam, Amsterdam, Netherlands; 7 Cancer Center Amsterdam, Amsterdam, Netherlands

**Keywords:** Liver, Cyst, Radiology, Surgery, Pathology

## Abstract

**Purpose:**

Mucinous cystic neoplasms of the liver (MCN-L) are hepatic cysts with a low malignant potential. The recent European Association for the Study of the Liver (EASL) guidelines provide guidance on the imaging features and surgical management of MCN-L, yet are hampered by a lack of studies adhering to the revised World Health Organization (WHO) criteria. This study attempted to validate the new 2022 EASL-guidelines in a retrospective cohort study of patients who underwent surgery for suspected MCN-L.

**Methods:**

Patients undergoing surgery for suspected MCN-L in a single center between 2010 and 2020 were included. Imaging features were assessed according to the EASL guidelines and were compared to final pathological diagnoses, according to the WHO criteria.

**Results:**

In total, 35 patients were included. In three patients, there were no worrisome imaging features, yet final pathological diagnosis showed MCN-L. Contrarily, six patients with worrisome imaging features did not have MCN-L. Five patients were diagnosed with MCN-L on final pathology. The sensitivity of the EASL-guidelines for the diagnosis of MCN-L was 40% (95%CI: 5.3–85%) and the specificity was 80% (95% CI: 61–92%).

**Conclusion:**

Although the new EASL-guidelines provide some guidance, they could not reliably distinguish MCN-L from other cysts in our series. Thus, preoperative diagnosis of MCN-L remains challenging and we should be careful in selecting surgical strategies based on these criteria.

## Introduction

Hepatic cysts are increasingly discovered due to a growing use of radiological imaging [1, 2]. Mucinous cystic neoplasms of the liver (MCN-L) represent less than 5% of (incidentally diagnosed) hepatic cysts which leads to various diagnostic and therapeutic challenges [3]. First, the (radiological) differentiation between MCN-L and simple hepatic cysts (SHCs), especially those with hemorrhage or cyst infection, is difficult [4, 5]. Second, MCN-L are assumed to have malignant potential, although the exact risk of malignant transformation remains unclear [6, 7]. These factors may lead to the undertreatment of patients with MCN-L by aspiration and sclerotherapy or deroofing, potentially leading to cyst recurrence and tumor seeding [8, 9]. Contrarily, patients with simple hepatic cysts without a risk of malignant transformation would be overtreated by radical (major) resection, with associated risks of morbidity and –although rare- even mortality [8, 10–12].

The formulation of clear recommendations on these challenges associated with MCN-L has been hampered by a lack of studies adhering to the revised WHO classification. In the 2010 WHO classification, the diagnosis of MCN-L is defined by presence of ovarian-like stroma showing positivity for estrogen receptor (ER) and progesterone receptor (PR) [6, 13–17]. Recently, the first guidelines adhering to the revised WHO classification were formulated by the European Association for the Study of the Liver (EASL). These guidelines state that complete surgical resection should be considered for suspected MCN-L, defined as cysts with one or more major and one or more minor worrisome imaging features. [18]

Nonetheless, the guidelines are based on a limited number of studies including only few MCN-L patients [19–21]. Thus, the aim of the current study was to validate the new 2022 EASL-guidelines in a retrospective cohort study of patients who underwent surgery for suspected MCN-L.

## Material and methods

### *Study design*

A retrospective cohort study was conducted adhering to the STrengthening the Reporting of OBservational studies in Epidemiology (STROBE) guidelines [22]. The medical ethical committee of the Amsterdam UMC declared that the study was beyond the scope of the Medical Research Involving Human Subjects Act (reference numbers location Academic Medical Center and location VU University Medical Center: 21.055 and 2021.0316, respectively). The study was conducted according to the Declaration of Helsinki. In line with the European General Data Protection Regulation (GDPR), patients who met the inclusion criteria were approached by their treating physician with a letter explaining the study goals and (data) procedures. All participants provided consent for the use of their data in the current study.

### Settings and subjects

A search engine for pseudonomyzed, unstructured data, CTcue (CTcue B.V., Amsterdam, The Netherlands), was used to search electronic patient files, including surgery and pathology reports, using the keywords ‘liver’ and ‘cyst’, including synonyms and Dutch translations. The search results were screened by two reviewers (AF and HS). All adult patients who underwent surgery for suspected MCN-L (or “cystadenoma”) between 2010 and 2020 in the Amsterdam UMC were included. Patients of whom pre-operative CT or MRI scans, pathology results, surgery details and/or outcomes were not available were excluded.

### Data collection and definitions

Pre-operative CT and MRI scans were reviewed according to the 2022 EASL-guidelines on cystic liver disease by a radiologist (OvD). The radiologist reviewed if scans were of sufficient quality for assessment, and was blinded for the final pathological diagnosis. Patients were classified as having high or low suspicion of MCN-L based on the presence or absence of worrisome features (Table 1) [18, 19]. Other imaging characteristics were also assessed (Supplementary File 1).

**Table 1:** 2022 EASL-criteria for high versus low suspicion of MCN-L

High suspicion of MCN-L	≥ 1 major and ≥ 1 minor feature
Low suspicion of MCN-L	Less than ≥ 1 major and ≥ 1 minor feature
Major features	Minor features
Nodularity	Upstream biliary dilatation
Thick septations^a^	Thin septations^a^
	Internal hemorrhage
	Perilesional perfusional change
	< 3 co-existent hepatic cysts

a Thick septations were defined as > 3 mm, and thin septations as < 3 mm in this study. Adapted from Drenth et al.[18]

**Fig. 1:**
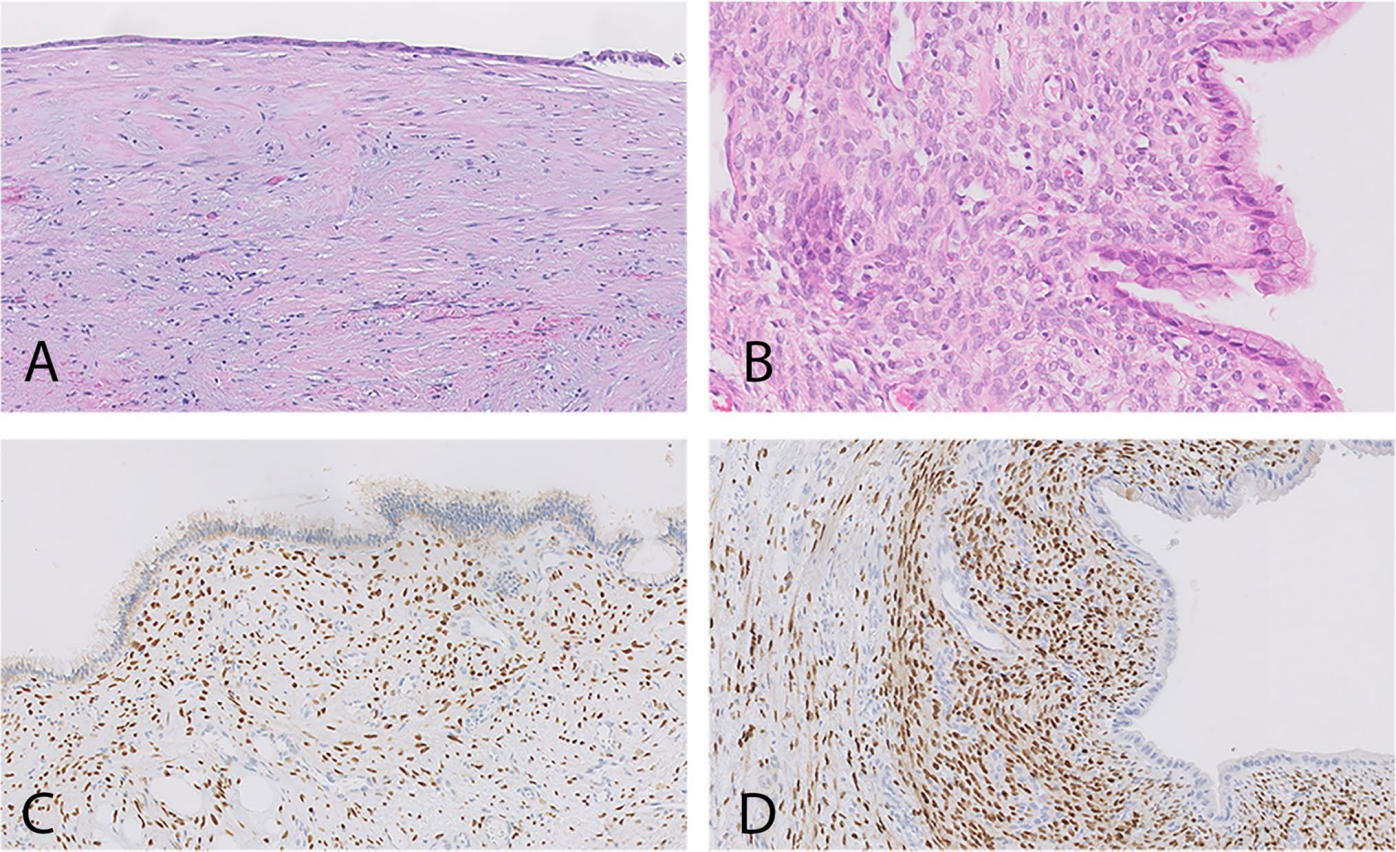
Representative slides of a simple hepatic cyst and mucinous cystic neoplasm of the liver A. Simple hepatic cyst, B-D. Mucinous cystic neoplasm of the liver with ovarian-like stroma and positive estrogen receptor (**C**) and progesterone receptor (**D**) staining

Pathology reports were consulted for the final pathological diagnosis. Next, histological slides (hematoxylin and eosin [H&E]) and additional estrogen receptor (ER) and progesterone receptor (PR) staining of surgical specimens of all patients with simple hepatic cysts or MCN-L were revised by an expert pathologist (JV). Hepatic cysts were classified as MCN-L if morphologically, (focal or diffuse) ovarian-like stroma was present with strong ER and/or PR staining. Figure 1 shows representative slides of a simple hepatic cyst and a MCN-L.

Clinical data were collected using the clinical data collection program Castor version 1.6 (Ciwit B.V., Amsterdam, the Netherlands). Relevant baseline characteristics were age, gender, body mass index (BMI), previous treatments and symptoms. Surgical outcomes were length of hospital stay (in days), blood loss (in mL), operating time (in minutes) and postoperative complications within 30 days (scored according to the Clavien-Dindo classification), and if complications occurred, the type of complication (f.e. bile leakage, postoperative hemorrhage, postoperative liver failure) [10]. Other outcomes were recurrence and mortality. When present, time to recurrence or mortality, presence of symptoms associated with recurrence, treatment of recurrence, and cause and date of death were recorded. Regarding the surgical procedures, the American Society of Anesthesiologists (ASA) score and type of surgical procedure (deroofing or complete resection, either minor or major resection) and approach (minimally invasive, yes or no) were recorded.

### Data analysis

In order to evaluate the diagnosis of patients with suspected MCN-L according to the EASL-guidelines, pathological diagnoses of patients with high suspicion of MCN-L on imaging (i.e. one or more major and one or more minor worrisome features) and patients with low suspicion of MCN-L on imaging were assessed. A detailed description was provided for patients with high suspicion of MCN-L on imaging, but with no MCN-L on pathology; as well as for patients with low suspicion of MCN-L on imaging, but with MCN-L on final pathology.

In addition, baseline characteristics and outcomes of deroofing and complete, minor resection were compared. Continuous data were reported as medians with their interquartile range (IQR), and compared using Mann–Whitney-U tests. Dichotomous variables and categorical data were reported as numerators and denominators, and compared using either chi-square tests or Fisher’s exact tests, as appropriate.

## Results

### Baseline characteristics

A total of 35 patients were included, with a median age of 55 years (IQR: 48–65), of whom four were male. Eight patients had previously undergone treatment for liver cysts, five had aspiration without sclerotherapy and three had aspiration combined with tetracyclin sclerotherapy. Symptoms were present in 27 patients, most commonly pain and/or discomfort (24 patients), palpable mass or bloating (seven patients), or difficulties eating (nausea, appetite loss and weight loss, six patients). Their median BMI was 25 kg/m^2^ (IQR: 21–27).

### Diagnosis according to EASL-guidelines

**Fig. 2:**
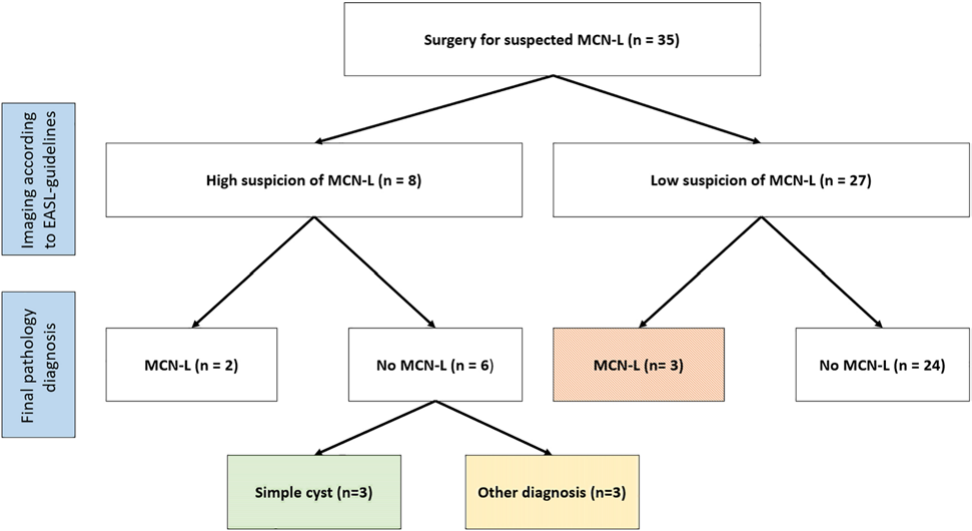
Application of the EASL criteria in our (high risk) population Orange (diagonal pattern filling) indicates patients that would be undertreated according to EASL-guidelines. Characteristics of these patients are shown in Table 2. Green (horizontal pattern filling) and yellow (vertical pattern filling) indicates patients that would be overtreated according to EASL-guidelines. Characteristics of these patients are shown in Table 3 and Supplementary Table 1, respectively

Fourteen patients had only CT available, eight only MRI and 13 had both. Eight out of 35 patients had high suspicion of MCN-L (i.e. a combination of one major and one minor worrisome feature) on imaging according to the 2022 EASL-guidelines. In total, five patients were diagnosed with MCN-L on final pathology, none of which showed signs of malignant transformation of MCN-L. Figure 2 shows the correlation between the imaging according to the EASL-guidelines and final pathological diagnosis. Sensitivity of the EASL-guidelines for diagnosing MCN-L on imaging was 40% (95%CI: 5.3–85%) and specificity was 80% (95% CI: 61–92%). The positive predictive value was 25% (95%CI: 8.2–54%) and the negative predictive value was 89% (95%CI: 80%-94%).

**Table 2:**
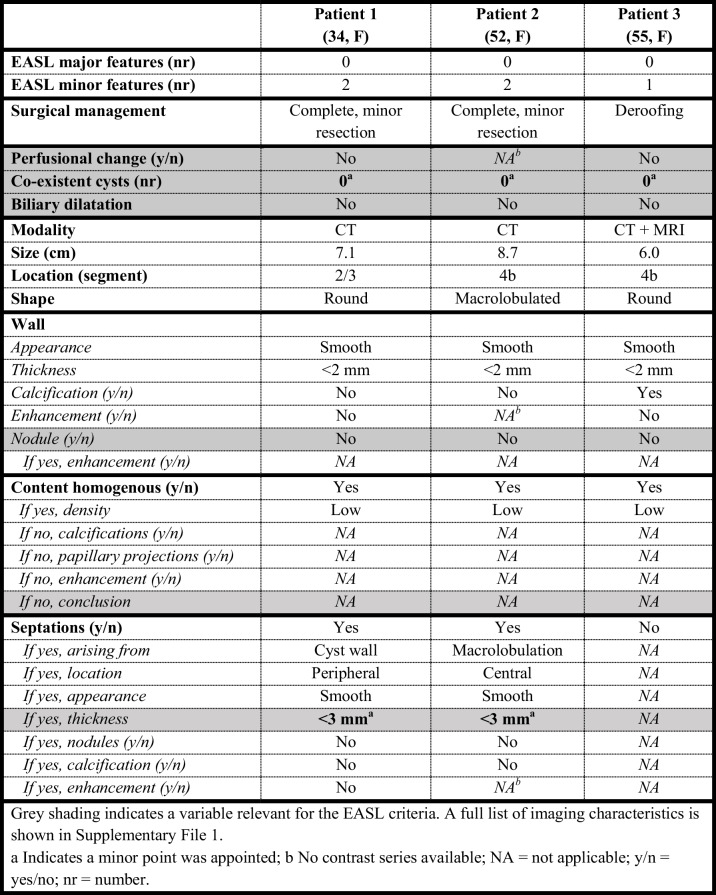
Patients with a low suspicion of MCN-L according to EASL-criteria, with a final diagnosis of MCN-L (orange category)

Grey shading indicates a variable relevant for the EASL criteria. A full list of imaging characteristics is shown in Supplementary File 1

^a^Indicates a minor point was appointed; b No contrast series available; NA = not applicable; y/n = yes/no; nr = number

**Table 3:**
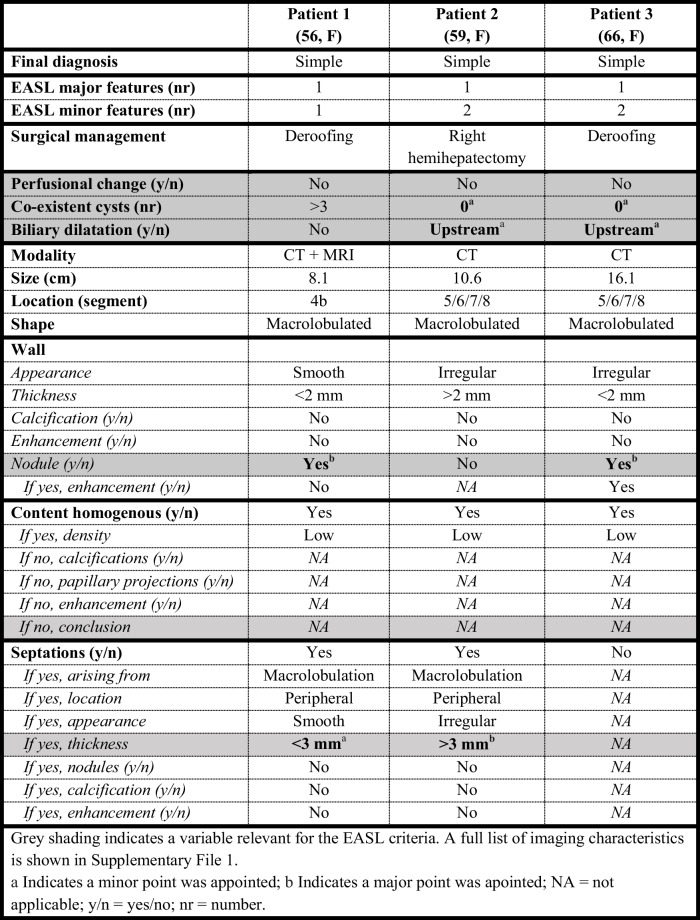
Patients with a high suspicion of MCN-L according to EASL-criteria, with a final diagnosis of simple cyst (green category)

Grey shading indicates a variable relevant for the EASL criteria. A full list of imaging characteristics is shown in Supplementary File 1

^a^Indicates a minor point was appointed; b Indicates a major point was apointed; NA = not applicable; y/n = yes/no; nr = number

**Fig. 3:**
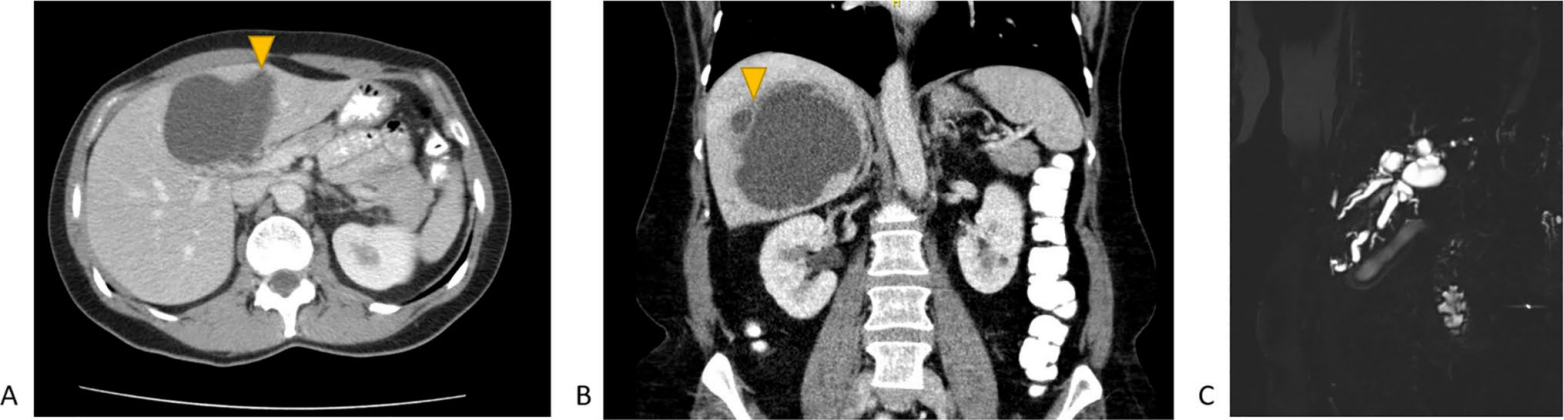
Representative CT and MRI images of patients in whom imaging assessment according to the EASL-guidelines did not correspond to the final pathological diagnosis A. CT-scan of a 52-year-old female patient with a liver cyst with low suspicion of MCN-L based on imaging assessment according to the EASL-guidelines. Final pathological diagnosis was MCN-L. B and C. CT-scan and MRI images of a 59-year-old patient with a liver cyst with high suspicion of MCN-L based on imaging assessment according to the EASL-guidelines. Final pathological diagnosis was simple hepatic cyst. A. The cyst measuring 8.7 cm, located in segment 4b, showed no nodularity or thick septations (no major worrisome features). Thin septations and less than three co-existent cysts were seen (two minor worrisome features). No upstream biliary dilatation, haemorrhage, or perilesional perfusional change were seen. B and C. The cyst measuring 10.6 cm in diameter, located in the right hemiliver, showed thick septations (one major worrisome feature), indicated by the yellow arrow. The cysts showed upstream biliary dilation (figure C) and less than three co-existent hepatic cysts (two minor worrisome features). No perilesional perfusional change or haemorrhage was observed

Six patients who had high suspicion of MCN-L on imaging did not have MCN-L on pathology. Five out of these six patients had no co-existent cysts as a minor worrisome feature on imaging. Three patients had simple cysts as final pathological diagnosis (Table 2). Three patients had alternate diagnoses on pathology, namely adenoma, endometriosis and serous cystadenofibroma (Supplementary File 2). Upstream biliary dilatation was seen on imaging in two of the three patients with simple hepatic cysts. Thick septations and solid intracystic content were observed on imaging in the patients with pathology diagnoses of adenoma and the patient with serious cystadenofibroma. Contrarily, three out of 27 patients with low suspicion of MCN-L on imaging had a final pathology diagnosis of MCN-L (Table 3). Two patients of these patients had cysts located in segment 4b on imaging. CT and MRI images of two patients in whom imaging assessment according to EASL-guidelines did not correspond to their final pathological diagnoses are shown in Fig. 3.

### Surgical outcome

Surgical procedures performed were deroofing (n = 15), complete, minor resection (n = 17) and complete, major resection (n = 3). Baseline characteristics and outcomes of deroofing and complete, minor resection are compared in Table 4. No significant differences were found between patients who underwent deroofing and patients who underwent complete, minor resection. Patients undergoing complete, minor resection tended to be younger (median 51 years) than patients undergoing deroofing (median 58 years). The majority of the deroofing and complete, minor resection procedures (11/15 and 9/17, respectively), were minimally invasive. More recurrences occurred after deroofing (n = 4/15) compared to complete, minor resection (n = 0/17, p = 0.038). Four patients who underwent deroofing had a radiological recurrence, two of whom had symptoms which required further treatment by percutaneous aspiration and sclerotherapy at approximately 6 months and 2 years after initial treatment. Three patients underwent complete, major resection (Supplementary File 3). Complications requiring postoperative drainage occurred in two patients, who had a hospital stay of 7 and 11 days. The other patient had no complications and a hospital stay of 7 days.

**Table 4:** Baseline characteristics and outcomes of deroofing versus complete, minor resection

	Deroofing (n = 15)	Complete, minor resection (n = 17)	p-value
Baseline characteristics			
Age at operation	58 (53–66)	51 (42–64)	0.079
ASA-score			0.449
*1*	5	3	
*2*	8	12	
*3*	2	1	
*Missing*	0	1	
Minimally invasive approach (%)	11 (73.3%)	9 (52.9%)	0.291^a^
Outcomes			
Hospital stay (days), median (IQR)	3 (3–7)	6 (4–7)	0.195
Blood loss (mL), median (IQR)	50 (10–231)^6^	75 (23–400)^11^	0.678
Operation time (min)	95 (63–149)^4^	127 (103–181)^3^	0.208
Complications			0.762
*Grade 1*	1	2	
*Grade 2*	0	0	
*Grade 3*	1	2	
Final diagnosis of MCN-L (yes)	1	4	0.338^a^
Follow-up (months), median (IQR)	3 (0–20)	4 (1–22)	0.455
Recurrence	4	0	0.038^a^
Mortality	0	0	1.000^a^

a Fisher’s exact test. Superscript number indicate the number of patients with missing data. IQR = interquartile range.

## Discussion

In this retrospective cohort study 35 patients who underwent surgery for suspected MCN-L were included. In nine of these patients, imaging assessment according to the 2022 EASL guidelines did not correlate with the final pathological diagnosis. This may be a related to the high risk (surgical) population in our study, in which five out of 35 patients had MCN-L. However, this study may also very well provide a reflection of the real-world situation, in which patients with cysts without suspicion of MCN-L are treated conservatively or by aspiration and sclerotherapy, and the guidelines are applied in high risk populations only. [8]

Complete resection of suspected MCN-L should be aimed for according to current guidelines. However, we showed that despite adherence to the guidelines, suspected MCN-L could not reliably be distinguished from simple hepatic cysts on imaging. There is currently no high-level evidence on reliable tumor markers [23]. Moreover, the exact risk of malignant transformation of MCN-L is unclear. [6, 7] Thus, surgical outcomes (associated with the extent of resection necessary to obtain a complete resection) are perhaps the most important factor on which treatment decisions should be based, in light of the high risk of misdiagnosis and unclear risk of malignant transformation.

In our study, complete, major liver resection was performed in three patients with a final diagnosis of simple cysts. The procedure was associated with grade 3 complications in two out of these three patients. More postoperative complications and longer postoperative hospital stay are observed after major liver resection, compared to minor liver resection [24]. Therefore, it is questionable if the benefits of performing a major liver resection outweigh the risks of misdiagnosis and operative complications.

No significant differences were found in terms of postoperative complications or hospital stay after deroofing or complete, minor resection, which is in accordance with the findings of Gall et al*.* [12] However, more recurrences were observed after deroofing (4/15 patients, of which two requiring further treatment) than after complete, minor resection (0/17 patients). These findings may support complete, minor resection rather than deroofing.

This study identified a set of alternate, infrequently occurring diagnoses that may also further hamper appropriate pre-operative diagnosis of MCN-L. In case of solid intracystic component, one should be aware of alternate diagnoses such as hepatocellular adenoma and serous cystadenofibroma. Hepatic endometriosis is rare, yet may also mimic MCN-L on imaging. [25]

Three MCN-L were missed by the risk stratification based on the guidelines. It is noteworthy that two of these three MCN-L were located in segment 4. Previous studies have described that 54% of MCN-L were located in segment 4 [14]. In addition, in the study by Anderson et al*.*, on which the recommendations of the EASL-guidelines seems largely based, a location in the left hemiliver was associated with MCN-L, albeit not significantly (p = 0.07) [19]. This suggests it may be helpful to include the cyst location in the pre-operative risk stratification, for example as a minor worrisome feature.

The results of our study should be interpreted in the light of limitations. First, long-term follow-up was lacking due to the retrospective nature of the study. This could, for example, have resulted in an underestimation of the risk of recurrence. Second, because MCN-L are rare cystic lesions of the liver, only five patients with MCN-L were included. All studies thus far using the revised WHO criteria have included less than thirty patients each [19–26]. A strength of our study was that patients were identified and included based on the pre-operative suspicion of MCN-L. The challenging differentiation with simple hepatic cysts is widely recognized, but this allowed alternate diagnoses to be recognized as an additional pitfall.

Future studies should preferably be large, international studies assessing (the correlation between) multiple imaging features and pathological diagnoses adhering to the WHO classification, with formal statistical hypothesis testing. In addition, more evidence on the risk of malignant transformation of MCN-L seems necessary, as the current study did not include any patients with signs of malignant transformation. A prospective design would be necessary to ensure adequate follow-up, but conducting a prospective study on the topic would be extremely challenging due to the rarity of MCN-L. Moreover, the process of malignant transformation may take years.

## Conclusion

In conclusion, we found that the new EASL-guidelines give some guidance. However, adequate preoperative diagnosis of MCN-L remains challenging and is not specific enough to guide clinical decision making, in particular the choice to resort to major surgery, observation or less invasive symptomatic treatment (i.e. aspiration and sclerotherapy or deroofing) in case of symptoms.

## Data Availability

The data that support the findings of this study are available from the corresponding author, upon reasonable request. Due to the nature of the research, due to privacy/ethical reason, some supporting data is not available.
